# Decoding Motor Skills: Video Analysis Unveils Age-Specific Patterns in Childhood and Adolescent Movement

**DOI:** 10.3390/children11111351

**Published:** 2024-11-05

**Authors:** Luca Russo, Massimiliano Micozzi, Ghazi Racil, Alin Larion, Elena Lupu, Johnny Padulo, Gian Mario Migliaccio

**Affiliations:** 1 Department of Theoretical and Applied Sciences, eCampus University, 22060 Novedrate, Italy; 2 Department of Biotechnological and Applied Clinical Sciences, Università dell’Aquila, 67100 L’Aquila, Italy; 3 Research Unit (LR 23JS01) “Sport Performance, Health & Society” Higher Institute of Sport and Physical Education of Ksar Said, Tunis 1000, Tunisia; 4 Faculty of Physical Education and Sports, Ovidius University of Constanta, 900470 Constanta, Romania; 5 Department of Motor Activities, Petroleum Gas University Ploiesti, 100600 Ploiesti, Romania; 6 Department of Biomedical Sciences for Health, Università degli Studi di Milano, 20133 Milan, Italy; 7 Department of Human Sciences and Promotion of the Quality of Life, San Raffaele Rome Open University, 00166 Rome, Italy; 8 Maxima Performa, Athlete Physiology, Psychology, and Nutrition Unit, 20126 Milan, Italy

**Keywords:** video analysis, gross motor skills, motor development, motor competence, kinematics

## Abstract

Motor skill development is crucial in human growth, evolving with the maturation of the nervous and musculoskeletal systems. Quantifying these skills, especially coordinative abilities, remains challenging. This study aimed to assess the performance of five motor tasks in children and adolescents using high-speed video analysis, providing data for movement and health professionals. Seventy-two volunteers were divided into three age groups: 27 first-grade primary school students (19 males and 8 females, aged 6.5 ± 0.5 years), 35 fourth-grade primary school students (16 males and 19 females, aged 9.2 ± 0.4 years), and 28 s-year middle school students (16 males and 12 females, aged 13.0 ± 0.3 years). Participants performed five motor tasks: standing long jump, running long jump, stationary ball throw, running ball throw, and sprint running. Each task was recorded at 120 frames per second and analyzed using specialized software to measure linear and angular kinematic parameters. Quantitative measurements were taken in the sagittal plane, while qualitative observations were made using a dichotomous approach. Statistical analysis was performed using the Kruskal–Wallis and Mann–Whitney tests with Bonferroni correction. Significant differences were observed across age groups in various parameters. In the standing long jump, older participants exhibited a longer time between initial movement and maximum loading. The running long jump revealed differences in the take-off angle, with fourth-grade students performing the best. Ball-throwing tests indicated improvements in the release angle with age, particularly in females. Sprint running demonstrated the expected improvements in time and stride length with age. Gender differences were notable in fourth-grade students during the running long jump, with females showing greater knee flexion, while males achieved better take-off angles. Video analysis effectively identified age-related and gender-specific differences in motor skill performance. The main differences were measured between first-grade primary school and second-year middle school students while gender differences were limited to all age groups. This method provides valuable insights into motor development trajectories and can be used by professionals to objectively assess and monitor the technical aspects of motor skills across different age groups.

## 1. Introduction

The study of motor skills is a crucial aspect for professionals and researchers involved in movement, especially when working with individuals of a developmental age. The developmental age, which spans from early childhood (2–7 years) through middle childhood (8–11 years) and adolescence (12–18 years) [1], represents a crucial period for motor skill development and acquisition. Motor skills are often very difficult to quantify, particularly when the focus is on coordinative motor skills. It is now well known that the development of motor skills changes with the maturation of the nervous, musculoskeletal systems, and with the changes in the hormonal structure of the individual’s body. In the literature, it is possible to find scientific works that objectify this evolution by describing it and offering insights and reference values [2].

Motor competence is strongly associated with both health [3] and academic performance [4] in children undergoing development. At the same time, it is well recognized in the literature that growth and physical development lead to a series of changes in motor skills [5,6]. In some instances, these changes may appear negative, with a temporary decline in motor competence, followed by subsequent improvement as growth progresses [7]. These developmental phases are delicate, and changes in motor competence and skills are often difficult to identify.

To avoid terminological confusion, the following section will analyze the definitions of motor skills and motor competence. Motor skills are defined as goal-directed movement patterns that are acquired and developed through practice [8]. These skills are traditionally categorized into two main groups: locomotor skills, such as running and jumping, and object control or manipulative skills, such as throwing or catching [9]. On the other hand, motor competence is a term that refers to the individual capacity to execute different motor acts, including the coordination of both fine and gross motor skills [10].

There are numerous qualitative tools for the assessment of movement skills, and the literature indicates that these are useful and valid methods for studying fundamental motor skills [11,12,13]. For example, the Test of Gross Motor Development (TGMD-3), which utilizes video analysis for assessment, has become a standard tool for evaluating fundamental movement skills in children [14]. At the same time, it remains challenging to find objective methods of analysis and evaluation that can quantify the motor characteristics of the movements being studied and analyzed. Among the most frequently examined movements for the assessment of motor skills and motor competence are running, jumping, and throwing [15]. These three types of movements are widely studied because they are present in many sports disciplines and play a significant role in the physical and motor development phases of children [10,16]. Although it is recognized that fundamental movement skills are more than run, jump, and throw [17], running, jumping, and throwing represent key fundamental motor skills as they are considered building blocks for more complex movement patterns. Running forms the basis for most locomotor activities. Jumping requires the coordination of multiple body segments and the integration of both horizontal and vertical forces. Throwing demonstrates advanced object manipulation and whole-body coordination [9]. A very simple method that can be used to objectify the quality of performed movements is video analysis [18]. Video analysis procedures are frequently and successfully used in the literature for the study of postural parameters [19,20], performance parameters [21], angular parameters [22], temporal parameters [23], and, more generally, to simplify kinematic measurements whenever the use of complex three-dimensional motion analysis is not feasible [24,25]. By combining this information and applying it to motor skills such as jumping, throwing, and running, it would be possible to better understand the motor behaviors of children during growth, with the aim of identifying potential delays in motor development.

This study hypothesizes that it is possible to identify the kinematic descriptors capable of providing quantitative information on the execution of three motor skills typical of children’s play phases: running, jumping, and throwing, with the latter two performed in multiple variants. These descriptors, applied to different and evolving age groups, would allow for a better understanding of how motor development influences children’s movement abilities. Therefore, this study aimed to assess the performance of five motor tasks in children and adolescents using high-speed video analysis, providing kinematic reference data for movement and health professionals. This approach can be considered a novelty in the assessment of movement skills.

## 2. Materials and Methods

### 2.1. Participants

For this cross-sectional experimental study, 99 participants were voluntarily recruited. Participants’ sampling was based on two local schools located in L’Aquila (Italy). For the participant’s recruitment, the help of Physical Education teachers interested in the project was fundamental. After the collection of the parents’ informed consent at the end of the tests, a total of 90 subjects were tested. The subjects were divided into the following groups: *n =* 27 first-grade primary school students (19 males and 8 females, aged 6.5 ± 0.5 years; stature 123.4 ± 6.1 cm; body mass 24.6 ± 5.3 kg), *n =* 35 fourth-grade primary school students (16 males and 19 females, aged 9.2 ± 0.4 years; stature 138.9 ± 6.4 cm; body mass 34.3 ± 6.6 kg), and *n =* 28 s-grade middle school students (16 males and 12 females, aged 13.0 ± 0.3 years; stature 159.7 ± 8.6 cm; body mass 47.1 ± 7.4 kg).

The selection of specific school grades was based on developmental stages described in the literature [26]. First-grade primary school, corresponding to students that are 6 years of age, represents early childhood, where fundamental motor patterns begin to emerge and stabilize. Fourth-grade primary school, corresponding to students that are 9 years of age, corresponds to middle childhood, a period characterized by rapid improvements in motor control and coordination. Second-grade middle school, corresponding to students that are 13 years of age, represents the onset of adolescence, marked by significant structural and hormonal changes that influence motor development [1]. This sampling strategy allowed us to examine motor skills across three crucial developmental phases [27].

The study was conducted in accordance with the Declaration of Helsinki, and it was approved by the local Research Ethics Committee under the number 631/2024.

### 2.2. Testing Procedures and Measurments

A total of 5 tests were performed: (1) Sprint run (SR); (2) Standing long jump (SLJ); (3) Long jump with run-up (SLJ-R); (4) Standing ball throw (BT); and (5) Ball throw with run-up (BT-R). The motor tasks were selected basing on the fundamental movement patterns that are usually evaluated in other validated motor assessment batteries, including the following: the TGMD-3 [14], the EUROFIT test battery [28], or the Movement Assessment Battery for Children (M-ABC) [29]. The testing procedures proposed in the present research were not a direct replication of standardized tests; they were designed aiming to capture the fundamental movement patterns in both static and dynamic conditions, allowing for a comprehensive analysis of motor behavior [30].

A kinesiologist [31,32] specialized in motor activities for developmental age (M.M.) supervised both the familiarization and the test sessions. The familiarization sessions were performed once a week for 3 weeks, and in the fourth week, the measurements were conducted using a video recording of the physical education session. All students understood the tests and completed all trials.

For video recording, a Casio Exilim EX-FH100 (Casio, Tokyo, Japan) camera set at 120 FPS was mounted on a tripod. Both the familiarization and test sessions were conducted using the same equipment and in the same gymnasium to ensure consistency. Additionally, the sequence of the trials followed the same order as previously described.

During the test session, each participant performed each individual trial twice. Only the second trial was video recorded and subsequently analyzed. Each trial had a specific setup, and all procedures were carried out with maximum safety precautions to minimize the risk of injuries.

After a proper calibration process, the video analysis measurements were performed using Kinovea V.0.9.5 (https://www.kinovea.org/, accessed on 30 August 2024), a free open source software frequently used in sport science research [21,22,33].

The video analysis procedures were conducted by another kinesiologist with extensive experience in video analysis (L.R.). Before proceeding with the final analysis, operator reliability assessment procedures were performed. After confirming that the measurement repeatability rate was greater than 90%, the definitive analysis of the video recordings was carried out.

#### 2.2.1. Sprint Run (SR)

**Figure 1:**
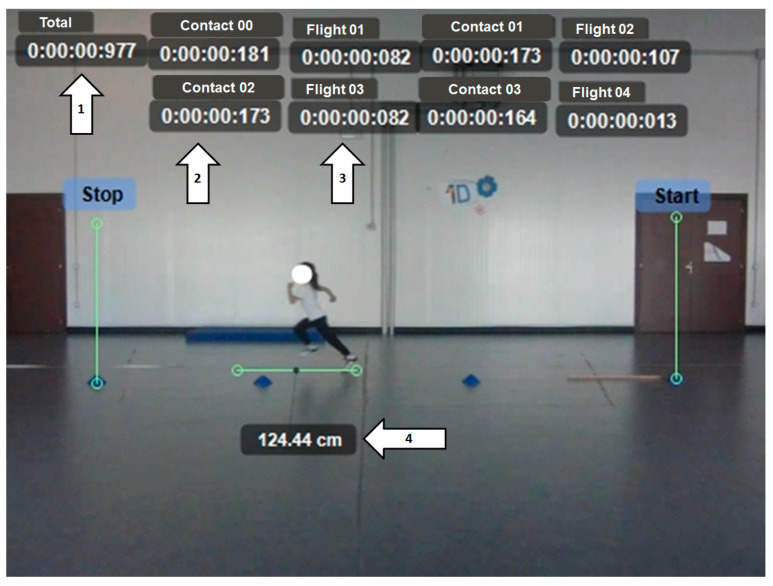
Sprint run (SR). 1: Running time to cover the central 6 m of the sprint. 2: Ground contact time. 3: Flight time. 4: Step length.

For this type of test (Figure 1), participants were instructed to run from a designated starting line to a finish line, which was set 18 m apart. The total distance was chosen based on the dimensions of the gymnasium that hosted the test sessions. The video recording captured the central 6 m of the run, thus excluding the start and acceleration phases as well as the finish and deceleration phases. A visual signal was used to indicate the start of the sprint, and participants were instructed to run as fast as possible in a straight line. For added safety, vertical mats were placed at the finish line to prevent collisions with the walls. For each subject, the following parameters were measured:

1. Running time to cover the central 6 m of the sprint;
2. Ground contact times;
3. Flight times;
4. Step length.

#### 2.2.2. Standing Long Jump (SLJ)

**Figure 2:**
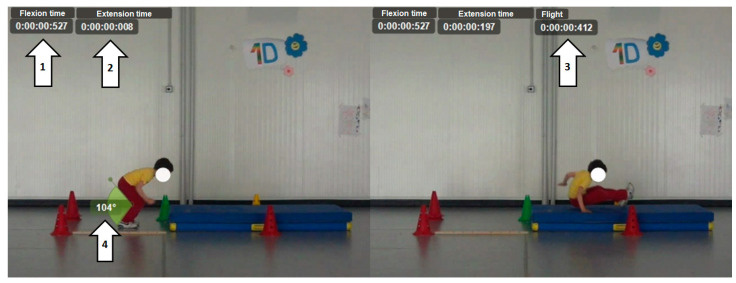
Standing long jump (SLJ). 1: Time from the initial movement to maximum knee flexion. 2: Time from maximum knee flexion to take-off. 3: Flight time. 4: Knee angle at maximum flexion.

For this type of test (Figure 2), participants were instructed to jump forward as far as they can without run-up. For added safety, participants landed on a mat to cushion the impact in case of a loss of balance after landing or an incorrect landing. For each subject, the following parameters were measured:

1. Time from the initial movement to maximum knee flexion (flexion time);
2. Time from maximum knee flexion to take-off (extension time);
3. Flight time;
4. Knee angle at maximum flexion;
5. Arm swing (Yes/No);
6. Take-off on one or two feet (1/2);
7. Leg tuck in the air (Yes/No).

The last three analyses of this movement were qualitative.

#### 2.2.3. Long Jump with Run-Up (SLJ-R)

**Figure 3:**
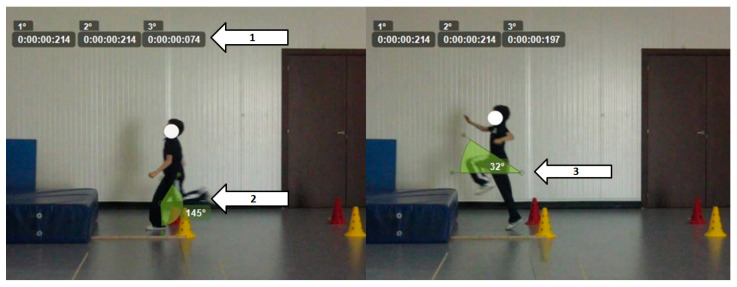
Long jump with run-up (SLJ-R). 1: Ground contact time of the last 3 steps. 2: Knee angle at maximum flexion. 3: Take-off angle.

In this test (Figure 3), participants were required to run for 5 m and then jump off one foot. The take-off point was unrestricted to avoid coordination influences associated with fixed distances. The parameters considered for this test did not account for the freedom in choosing the take-off point; therefore, the variability in jump length was not measured. For each subject, the following parameters were measured:

1. Ground contact time of the last 3 steps (only the last one was statistically analyzed);
2. Knee angle at maximum flexion (before take-off);
3. Take-off angle.

#### 2.2.4. Standing Ball Throw (BT)

**Figure 4:**
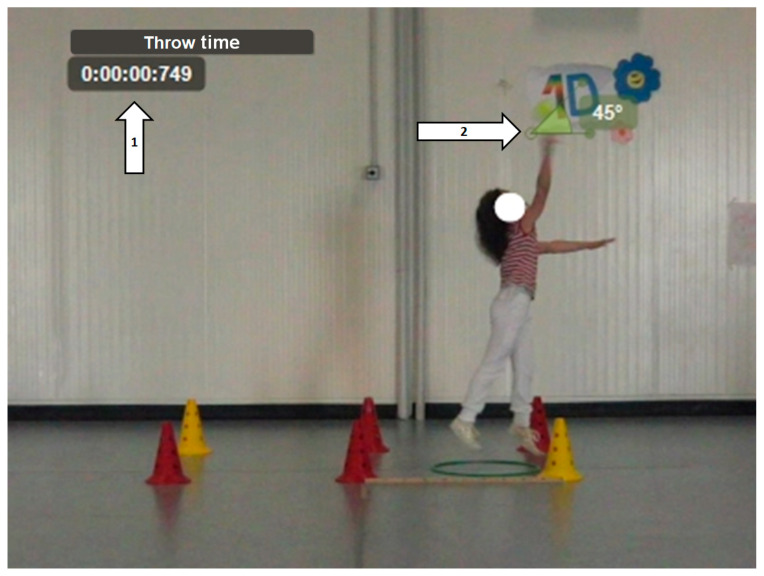
Standing ball throw (BT). 1: Time from maximum posterior loading to release of the ball. 2: Angle of the ball at release.

For the execution of this test (Figure 4), a softball was used. The size of the ball allowed it to be easily visible to the camera and comfortably grasped by all participants. Participants were required to keep their non-throwing arm (without the ball) in a forward-upward position, perform a loading phase, and then throw the ball as far as possible. Similarly to the jumping tests, performance measures of throw distance were not conducted; instead, the following parameters were evaluated:

1. Time from maximum posterior loading to the release of the ball (throw time);
2. Angle of the ball at release;
3. Support on one or two feet during the release (Yes/No);
4. Position of the opposite arm in relation to the shoulder during the release (above/below the shoulder).

The last two analyses of this movement were qualitative.

#### 2.2.5. Standing Ball Throw with Run-Up (BT-R)

**Figure 5:**
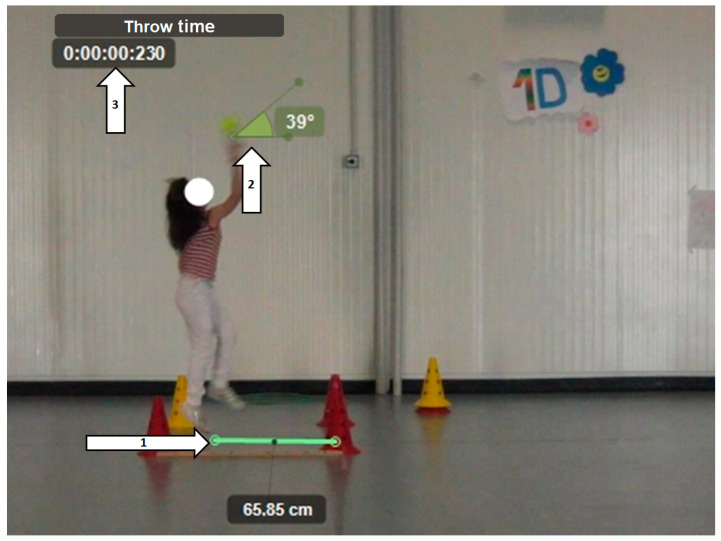
Standing ball throw with run-up (BT-R) 1: Horizontal distance between the front support foot and the throw line. 2: Angle of the ball at release. 3: Time from maximum posterior loading to release of the ball.

For this test (Figure 5), a softball was also used, but participants were required to throw the ball after a 5 m run-up. Unlike the other tests, a reference line was present from which to throw. The following parameters were evaluated:

1. Horizontal distance between the front support foot and the throw line (line distance);
2. Angle of the ball at release;
3. Time from maximum posterior loading to the release of the ball (throw time).

### 2.3. Statistical Analysis

Descriptive data are presented as the mean and standard deviation (SD). Shapiro–Wilk and Levene’s tests were used to verify normal distribution of the data and the homogeneity of variances, respectively. Due to certain anomalies in the normal distribution of some parameters causing a numerical difference in the sample divided for age and sex, it was decided that a fairer statistical analysis should be conducted using non-parametric tests. The Kruskal–Wallis test was employed for a first general comparison within trials, once it was significant, followed by the Mann–Whitney test with Bonferroni correction for comparisons between age groups. Gender differences in specific class ages were analyzed separately using the Mann–Whitney test with Bonferroni correction.

Statistical significance was set at *p* < 0.05. All analyses were performed with the Statistical Package for the Social Sciences (release 24.0, IBM^®^, Chicago, IL, USA).

## 3. Results

Data have been separated for gender in the analysis because of the well-known presence of gender differences during the developmental age [2,34]. Because of the relevant quantity of measured data, the results will be presented, dividing the quantitative and the qualitative assessment for each test.

### 3.1. Quantitative Data

The results of sprint run (SR) are presented in Table 1.

Contact time did not show any differences across the age groups both for males and females. The main differences were measured between first-grade primary school and fourth-grade primary school for flight time and step length (males: *p* = 0.000 and 0.000, respectively; females: *p* = 0.001 and 0.001, respectively) and between first-grade primary school and second-grade middle school for flight time and step length (males: *p* = 0.000 and 0.000, respectively; females: *p* = 0.010 and 0.002, respectively). Differences between the fourth-grade primary school and second-grade middle school were measured only for step length (males: *p* = 0.000; females: *p* = 0.000).

No significant differences were measured for this test between males and females.

The results of standing long jump (SLJ) are presented in Table 2.

Flight time and maximum flexion knee angle did not show any differences across the age groups both for males and females. Just one difference was present in the female group between fourth-grade primary school groups and second-grade middle school groups for flexion time (*p* = 0.002). While in the male group, two differences were present for flexion time between first-grade and fourth-grade primary school groups and second-grade middle school groups (*p* = 0.003 and 0.000, respectively). Another difference for the male group was measured in flight time between first-grade primary school and second-grade middle school (*p* = 0.002).

No significant differences were measured for this test between males and females.

The results of long jump with run-up (SLJ-R) are presented in Table 3.

The flight time and maximum flexion knee angle did not show any differences across the age groups both for males and females. Just one difference was present in the female group between fourth-grade primary school participants and second-grade middle school participants for flexion time (*p* = 0.002). In the male group, while two differences were present for flexion time between first-grade and fourth-grade primary school participants and second-grade middle school participants (*p* = 0.003 and 0.000, respectively). Another difference for male group was measured in flight time between first-grade primary school participants and second-grade middle school participants (*p* = 0.002).

**Figure 6:**
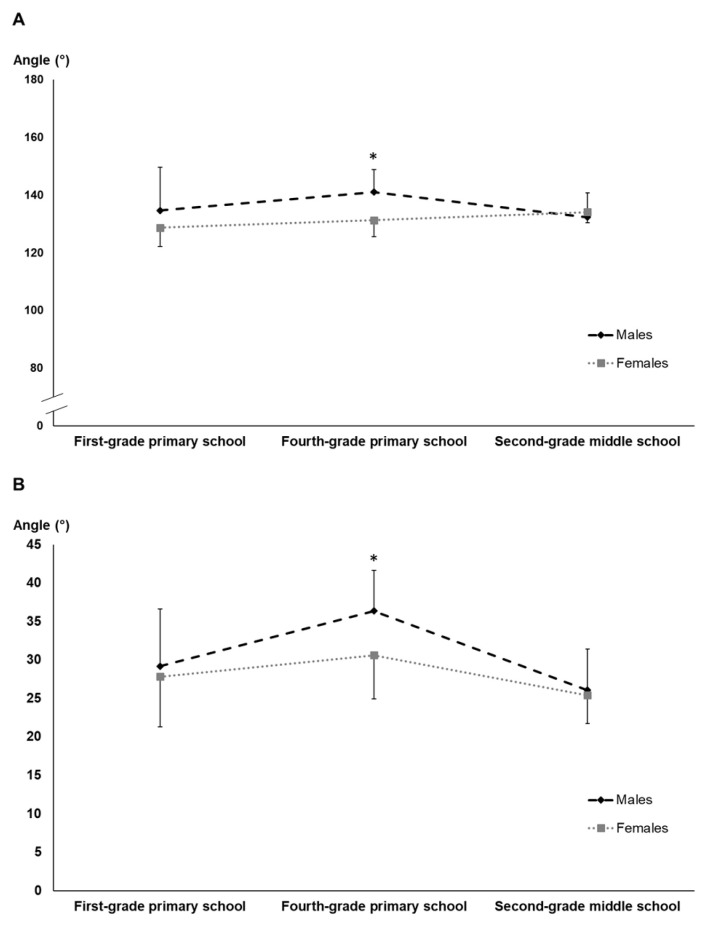
Differences in the long jump with run-up (SLJ-R) between genders according to age groups: (**A**) values for maximum flexion knee angle before the take-off; (**B**) values for take-off angle. Note. * Significant differences between genders in fourth-grade primary school participants.

Two significant differences were measured in this test between males and females only for the Fourth-grade primary school participants (Figure 6). The differences were measured for the maximum flexion knee angle before the take-off (males: 141.0 ± 7.8°; females: 131.2 ± 9.3°; *p* = 0.007) and for the take-off angle (males: 36.4 ± 5.3°, females: 30.6 ± 5.7°; *p* = 0.011).

The results of standing ball throw (BT) are presented in Table 4.

Standing ball throw (BT) showed the most stable results across the age groups, with just one difference being present for males between fourth-grade primary school participants and second-grade middle school participants for the release angle of the ball (*p* = 0.011).

No significant differences were measured for this test between males and females.

The results of the standing ball throw with run-up (BT-R) are presented in Table 5.

Standing ball throw with run-up (BT-R) showed a stable behavior across the age groups as well as BT. No statistical differences were measured for males and only two statistical differences were measure for females in the throw time between first-grade and fourth-grade primary school participants and between first-grade primary school participants and second-grade middle school participants (*p* = 0.003 and *p* = 0.000, respectively).

No significant differences were measured for this test between males and females.

### 3.2. Qualitative Data

Qualitative evaluation was performed for two tests: SLJ and BT. The aim of the qualitative assessment was to describe the motion quality.

The parameters assessed using video and the relative key of interpretations are presented in Table 6. The behavior was assessed as “positive” in the case the participant followed the kinesiologist’s prescription of the movement. In case the movement was performed with a different form, the behavior was assessed as “negative”.

The results for the qualitative assessment of SLJ are presented in Table 7.

The results for the qualitative assessment of BT are presented in Table 8.

## 4. Discussion

The principal aim of this study was to compare the motor behavior of five basic motor tasks in children and adolescents, providing kinematic preliminary data for movement and health professionals. The research was led hypothesizing that it is possible to identify the kinematic descriptors capable of providing quantitative information on the execution of three motor skills such running, jumping, and throwing.

Previous studies have primarily focused on standardized test batteries for assessing motor skills in children. D’Isanto et al. [35] used the MABC-2 to assess motor skills in 5–6-year-old children. Their research focused on manual dexterity, ball skills, and balance skills through standardized scoring systems. Similarly, a systematic review by Scheuer et al. [13] identified different motor test instruments that measure either motor abilities, motor skills, motor competencies through normative or criterion-referenced approaches. The main novelty of the present research lies in its specific methodological approach. While previous studies have primarily focused on measuring the performance outcomes, the present study used high-speed video analysis to examine both the quantitative and qualitative aspects of movement execution, without regard for the performance outcomes. This approach has the potential to provide a more comprehensive understanding of motor development patterns across different age groups, analyzing the specific kinematic parameters such as contact times, knee angles, and temporal phases of movements [13,35]. The use of video analysis allows for both the qualitative and quantitative assessment of movement execution, focusing on the technical aspects of movement and not on pure performance aspects, the latter are continuously changing during the developmental age, and therefore its assessment could be not useful.

Before discussing the results, it is worth noting that the interpretation of motor skill differences during the first years of primary school requires careful consideration. During this age, the motor skill proficiency may be more mainly influenced by individual developmental trajectories and/or previous movement experiences rather than chronological age alone [36]. In fact, during early childhood, the motor skill development can be highly variable [37]: some children show advanced motor patterns while others are still developing basic movement competencies. Various factors including environmental influences, previous physical activity experiences, and biological maturation rates may generate these individual differences [38].

The overall analysis of the results confirms the initial hypothesis: kinematic descriptors obtained through video analysis can be used to effectively identify age-related differences in movement execution. The data patterns generally aligned with the authors’ expectations. Improvements are showed across age groups but with interesting variations in the rate of development across different motor skills. The most interesting aspect is that while traditional performance-based tests typically show linear improvements with age, the kinematic analysis revealed a brand new picture of motor development. The principal examples are running and throwing. Running parameters showed a clear progression across age groups, particularly in step length and flight time; in contrast, throwing patterns demonstrated more stability across age groups, with only minor variations in release angles. Running evolution suggests a relatively rapid development, while throwing stability indicates a slower developmental trajectory for this more complex motor skill. Once again, these findings suggest that a specific kinematic analysis can help in capturing some specific aspects of motor development that might not be evident when measuring only performance outcomes.

Going into the specific results of this study, the sprint run (SR) test data can be discussed. The SR test is undoubtedly the most performance-driven among all those used in this research. It requires a reduced amount of coordinative control, counterbalanced by a greater physical demand. This aspect is confirmed by the trend of the measured parameters, which tend to improve with age. In line with previous literature [39,40], the running time significantly decreases, while both flight times and step length increase significantly. The latter shows a notable increase not only when comparing first-grade primary school with second-grade middle school students, but also when comparing first-grade and fourth-grade primary school students, confirming that anthropometric growth plays a fundamental role in this test [41]. On the other hand, the trend in the contact time is interesting. It never changes across the age group. The contact time relates to the step frequency both in running and in walking [42,43], and at the same time, it is connected with muscle and tendons stiffness as well [44]. For these reasons, the lack of changes in this parameter between age groups could be an indication of the students’ musculoskeletal system not yet having fully matured. It could also serve as a reference parameter to begin studying running technique among young individuals. Moreover, the stability across age groups in contact time might suggest that improvements in sprint performance are primarily driven by increases in step length and flight time rather than changes in ground contact mechanics. This aspect requires further investigation.

The standing long jump (SLJ) may appear to be a very simple task, but it challenges the coordination system and motor control, particularly in managing the simultaneous push-off from both feet in coordination with arm swing. For this reason, vertical jump tests are often preferred to reduce the coordinative component, focusing more on measuring performance [45]. From the qualitative analysis, it appears that a very high percentage of participants across all age groups manage this movement effectively. However, the quantitative analysis reveals some interesting aspects. The increased flexion time observed in second-grade middle school participants, particularly in males, could indicate a more developed ability to utilize the stretch-shortening cycle [46] or an increased difficulty in stabilizing the body during knee flexion, particularly for adolescents that are quickly growing up. On the other hand, no modifications occurred for extension time, suggesting that the explosive phase of the movement is stable for a very long time during developmental age.

The long jump with run-up (SLJ-R) was the only test to reveal intriguing gender differences, particularly in the fourth-grade primary school group. Males demonstrated better take-off angles, while females exhibited greater knee flexion. These differences align with previous research highlighting gender-specific motor skill development patterns [2,34]. The fact that fourth-grade students performed the best in terms of take-off angle suggests a potential “golden age” for motor skill development. It has been previously reported in the literature [47] that it is already being discussed among researchers. The significant reduction in the take-off angle in both male and female second-grade middle school participants can be read as another particular aspect of the complex phase that is puberty.

The throwing tests show the lowest rate of changes across age groups, confirming the complex nature of the throwing motor pattern, which requires many more years to learn and perfect it [48]. The stability observed in the standing ball throw (BT) across age groups, with only minor differences in release angle for males, suggests and confirms that this skill may develop more slowly or require specific training for significant improvements. The qualitative evaluation of the standing ball throw (BT) provides valuable insights into the proper development of the use of the non-throwing arm, which progressively improves in alignment during growth according to the instructions from the kinesiologist. There is also a gendered difference in the percentage of positive behaviors in managing foot positioning during the throw. Among males, this coordination tends to improve with age, while among females, it is generally better during primary school but declines in middle school, likely due to the onset of puberty, indicating a slight reduction in coordination skills during this developmental stage. Regarding the quantitative measurements taken during the standing ball throw with run-up (BT-R), the most interesting result is the measure of the distance from the throwing line. This measurement exhibits significant variability across all age groups. It can be considered an index of spatiotemporal coordination, which is crucial for determining where to stop before throwing the ball after a run-up. This parameter is particularly interesting and undoubtedly opens new scenarios for research aimed at simplifying the study of coordination skills, a topic that is inherently complex to investigate.

Before concluding the discussion, several additional factors that could influence the motor task are worth mentioning. Some of these factors may be environmental conditions during testing, such as surface type and testing environment (indoor vs. outdoor condition). Moreover, psychological factors such as motivation and anxiety can significantly influence the motor task execution [49].

While the present study focused on specific motor tasks that could be effectively analyzed through video analysis, it is important to remind that there are other fundamental motor abilities, such as balance, that have not been evaluated but would have provided a more comprehensive assessment of motor development. In fact, balance requires different assessment approaches beyond two-dimensional video analysis.

Finally, the sequential nature of testing may have allowed participants to observe and potentially imitate the movement patterns of previous performers. It opens the possibility of affecting the natural own execution of the movements for each participants. This “modeling effect” was documented a long time ago [50], although its impact on testing situations is not clear at all.

### Limitations

Although this study provides valuable insights into the development of motor skills using the kinematic video analysis as an innovative approach, it is not free of limitations.

Firstly, the cross-sectional design limits our ability to draw causal conclusions about motor skill development over time. Such limitations are typical of this kind of investigation method. A longitudinal study would provide more robust evidence.

Secondly, the sample size, particularly when divided by age and gender, is not so large. It may limit the generalizability of the findings, and therefore, future research including larger and more diverse samples are needed. Moreover, the relative age effect could also be a potential confounding factor in this study, since within each grade level, children can differ in age by up to 12 months, influencing motor development and performance as well [51,52]. In addition, body mass index (BMI) has been shown to influence motor performance patterns [53]. While not directly measured in this study, these factors represent important considerations for future research in motor development assessment.

Additionally, while the video analysis provided valuable quantitative and qualitative data, the two-dimensional nature of the analysis may have missed some aspects of three-dimensional movement. Two-dimensional video analysis is easy and it is an “evergreen” tool; however, in the near future, the use of video analysis will integrate artificial intelligence algorithms, allowing one to obtain three-dimensional data. This will help the analysis and the measures’ reliability of movement patterns.

Finally, the qualitative assessment, while informative, could benefit from a more standardized scoring system to enhance reliability and allow for more nuanced comparisons across groups.

Furthermore, the study did not account for factors such as physical activity levels, sports participation, or maturation status, which could influence motor skill development.

All these limitations could not be addressed within a single study, and they will serve as the basis for future investigations. Nonetheless, the current findings highlight the complex nature of motor skill development and underscore the importance of considering both quantitative and qualitative aspects of movement execution. This approach offers a promising method for movement and health professionals to objectively assess and monitor the technical aspects of motor skills in children and adolescents.

## 5. Conclusions

Kinematic video analysis can be considered a useful and cost-effective tool for evaluating motor skills but also provides unique insights into age- and gender-specific movement patterns. From a biomechanical perspective, the present findings revealed distinct developmental patterns. Sprint performance improvements were primarily driven by increases in step length and flight time rather than changes in ground contact mechanics. Jumping patterns showed age-related differences in flexion time and take-off angles, which were particularly evident in the fourth-grade group. Throwing mechanics demonstrated the most stability across the age groups, confirming its nature as a complex motor skill and requiring specific training for significant improvements.

Gender differences were most relevant in the running long jump among fourth-grade students, with males showing better take-off angles and females exhibiting greater knee flexion. The biomechanical differences could suggest the presence of gender-specific movement strategies.

The present findings contribute to the research field in three main ways. First, the presence of detailed kinematic references for different age groups can be used in future research and practical applications. Second, video analysis can capture subtle developmental differences in movement patterns that might be missed by traditional performance measures. Third, the findings highlight the importance of considering both age- and gender-specific movement characteristics in motor development assessment. Future studies should focus on expanding these findings with larger sample sizes and longitudinal designs.

## Figures and Tables

**Table 1:** Results for the qualitative measurements of the sprint run (SR) for the male and female participants of all age groups.

	Males (n = 51)			Females (n = 39)		
Variables	First-Grade Primary School	Fourth-Grade Primary School	Second-Grade Middle School	First-Grade Primary School	Fourth-Grade Primary School	Second-Grade Middle School
Running time (s)	1.42 ± 0.1	1.35 ± 0.2	1.24 ± 0.1 *	1.44 ± 0.1	1.39 ± 0.1	1.27 ± 0.1 *
Contact time (ms)	150.4 ± 19.3	146.9 ± 32.1	157.2 ± 22.9	155.9 ± 12.4	152.8 ± 20.0	149.4 ± 19.1
Flight time (ms)	88.4 ± 15.1	115.6 ± 19.5 §	124.1 ± 20.7 *	99.5 ± 18.6	129.9 ± 18.7 §	127.7 ± 26.3 *
Step lenght (cm)	101.1 ± 9.9	119.0 ± 12.2 §	149.7 ± 11.9 *#	107.7 ± 9.9	123.6 ± 8.9 §	143.4 ± 9.9 *#

Note. * Significant difference between first-grade primary school participants and second-grade middle school participants; § significant difference between first-grade and fourth-grade primary school participants; # significant difference between fourth-grade primary school participants and second-grade middle school participants. The differences in the table are shown within the gender groups.

**Table 2:** Results for the qualitative measurements of the standing long jump (SLJ) for male and female participants of all age groups.

	Male (n = 51)			Female (n = 39)		
Variables	First-Grade Primary School	Fourth-Grade Primary School	Second-Grade Middle School	First-Grade Primary School	Fourth-Grade Primary School	Second-Grade Middle School
Flexion time (ms)	649.3 ± 166.9	597.2 ± 169.1	1037.3 ± 423.8 *#	620.3 ± 129.7	487.6 ± 112.1	752.7 ± 240.6 #
Extension time (ms)	240.2 ± 39.5	221.9 ± 67.4	253.3 ± 74.8	222.6 ± 25.7	203.4 ± 54.7	244.4 ± 58.4
Flight time (ms)	381.4 ± 50.3	403.9 ± 63.9	447.8 ± 59.5 *	391.7 ± 20.3	329.7 ± 44.2	424.6 ± 61.5
Knee angle (°)	105.0 ± 10.8	114.5 ± 13.8	108.8 ± 9.0	97.9 ± 13.9	106.9 ± 12.4	101.4 ± 14.8

Note. * Significant differences between first-grade primary school participants and second-grade middle school participants; # significant difference between fourth-grade primary school participants and second-grade middle school participants. The differences in the table are shown within the gender groups.

**Table 3:** Results for the qualitative measurements of the long jump with run-up (SLJ-R) for male and female participants of all age groups.

	Males (n = 51)			Females (n = 39)		
Variables	First-Grade Primary School	Fourth-Grade Primary School	Second-Grade Middle School	First-Grade Primary School	Fourth-Grade Primary School	Second-Grade Middle School
Contact time (ms)	180.5 ± 31.0	200.7 ± 37.7	203.6 ± 23.5 *	194.0 ± 32.0	202.5 ± 33.9	200.6 ± 39.4
Knee angle (°)	134.6 ± 15.1	141.0 ± 7.8	132.3 ± 8.4 #	128.7 ± 11.4	131.2 ± 9.3	134.1 ± 7.3
Take-off angle (°)	29.2 ± 7.5	36.4 ± 5.3 §	26.1 ± 5.4 #	27.8 ± 6.5	30.6 ± 5.7	25.4 ± 3.7 #

Note. * Significant differences between first-grade primary school participants and second-grade middle school participants; § significant difference between first-grade and fourth-grade primary school participants; # significant difference between fourth-grade primary school participants and second-grade middle school participants. The differences in the table are showed within the gender groups.

**Table 4:** Results for the qualitative measurements of the standing ball throw (BT) for male and female participants of all age groups.

	Males (n = 51)			Females (n = 39)		
Variables	First-Grade Primary School	Fourth-Grade Primary School	Second-Grade Middle School	First-Grade Primary School	Fourth-Grade Primary School	Second-Grade Middle School
Throw time (ms)	266.7 ± 66.4	232.8 ± 56.6	259.0 ± 57.2	235.4 ± 77.2	254.0 ± 44.7	248.1 ± 69.5
Release angle (°)	36.3 ± 11.9	35.7 ± 8.1	28.8 ± 4.7 #	24.4 ± 10.6	29.3 ± 11.8	35.2 ± 13.3

Note. # significant difference between fourth-grade primary school participants and second-grade middle school participants. The differences in the table are showed within the gender groups.

**Table 5:** Results for the qualitative measurements of the standing ball throw with run-up (BT-R) for male and female participants of all age groups.

	Males (n = 51)			Females (n = 39)		
Variables	First-Grade Primary School	Fourth-Grade Primary School	Second-Grade Middle School	First-Grade Primary School	Fourth-Grade Primary School	Second-Grade Middle School
Line distance (cm)	−3.8 ± 60.9	−19.8 ± 46.5	−17.6 ± 32.9	23.5 ± 45.3	−8.9 ± 45.4	−30.7 ± 37.7
Throw time (ms)	239.6 ± 109.2	201.2 ± 49.6	204.7 ± 51.1	161.4 ± 47.7	182.0 ± 41.7 §	193.4 ± 32.1 *
Release angle (°)	28.6 ± 12.7	38.5 ± 10.7	33.6 ± 8.8	16.9 ± 6.2	31.5 ± 11.7	36.9 ± 8.8

Note. * Significant differences between first-grade primary school participants and second-grade middle school participants; § significant difference between first-grade and fourth-grade primary school participants. The differences in the table are showed within the gender groups.

**Table 6:** Key of interpretation for the qualitative assessment.

Variables	Positive Behavior	Positive Behavior	Negative Behavior
SLJ	Arm swing	Yes	No
	Take-off on one or two feet	2	1
	Leg tuck in the air	Yes	No
BT	Support on two feet during the release	Yes	No
	Position of the opposite arm in relation to the shoulder during the release	Above the shoulder	Below the shoulder

Note: Standing long jump (SLJ), standing ball throw (BT).

**Table 7:** SLJ qualitative assessment results.

	Males (n = 51)						Females (n = 39)					
Variables	First-Grade Primary School		Fourth-Grade Primary School		Second-Grade Middle School		First-Grade Primary School		Fourth-Grade Primary School		Second-Grade Middle School	
	Pos	Neg	Pos	Neg	Pos	Neg	Pos	Neg	Pos	Neg	Pos	Neg
Arm swing	83%	17%	93%	7%	100%	0%	100%	0%	100%	0%	91%	9%
Take-off on one or two feet	78%	22%	53%	47%	87%	13%	86%	14%	84%	16%	73%	27%
Leg tuck in the air	94%	6%	100%	0%	100%	0%	100%	0%	100%	0%	100%	0%

Note. Pos: positive behavior. Neg: negative behavior. %: percentage of participants that showed positive or a negative behavior.

**Table 8:** BT qualitative assessment results.

	Males (n = 51)						Females (n = 39)					
Variables	First-Grade Primary School		Fourth-Grade Primary School		Second-Grade Middle School		First-Grade Primary School		Fourth-Grade Primary School		Second-Grade Middle School	
	Pos	Neg	Pos	Neg	Pos	Neg	Pos	Neg	Pos	Neg	Pos	Neg
Support on one or two feet during the release	16%	84%	31%	69%	67%	33%	62%	38%	68%	32%	33%	67%
Position of the opposite arm in relation to the shoulder during the release	84%	16%	87%	13%	100%	0%	87%	13%	85%	15%	83%	17%

Note. Pos: positive behavior. Neg: negative behavior. %: percentage of participants that showed positive or a negative behavior.

## Data Availability

The data that support the findings of this study are available from the corresponding author upon reasonable request.
